# The field experiments and model of the natural dust deposition effects on photovoltaic module efficiency

**DOI:** 10.1007/s11356-018-1970-x

**Published:** 2018-04-20

**Authors:** Marek Jaszczur, Janusz Teneta, Katarzyna Styszko, Qusay Hassan, Paulina Burzyńska, Ewelina Marcinek, Natalia Łopian

**Affiliations:** 1 Faculty of Energy and Fuels, AGH University of Science and Technology, Krakow, Poland; 2 Faculty of Electrical Engineering, Automatics, Computer Science and Biomedical Engineering, AGH University of Science and Technology, Krakow, Poland; 3 Department of Mechanical Engineering, University of Diyala, Baqubah, Iraq

**Keywords:** PV efficiency loss, Photovoltaic modules, PV derating factor, Dust deposition, Renewable energy, Air pollution

## Abstract

The maximisation of the efficiency of the photovoltaic system is crucial in order to increase the competitiveness of this technology. Unfortunately, several environmental factors in addition to many alterable and unalterable factors can significantly influence the performance of the PV system. Some of the environmental factors that depend on the site have to do with dust, soiling and pollutants. In this study conducted in the city centre of Kraków, Poland, characterised by high pollution and low wind speed, the focus is on the evaluation of the degradation of efficiency of polycrystalline photovoltaic modules due to natural dust deposition. The experimental results that were obtained demonstrated that deposited dust-related efficiency loss gradually increased with the mass and that it follows the exponential. The maximum dust deposition density observed for rainless exposure periods of 1 week exceeds 300 mg/m^2^ and the results in efficiency loss were about 2.1%. It was observed that efficiency loss is not only mass-dependent but that it also depends on the dust properties. The small positive effect of the tiny dust layer which slightly increases in surface roughness on the module performance was also observed. The results that were obtained enable the development of a reliable model for the degradation of the efficiency of the PV module caused by dust deposition. The novelty consists in the model, which is easy to apply and which is dependent on the dust mass, for low and moderate naturally deposited dust concentration (up to 1 and 5 g/m^2^ and representative for many geographical regions) and which is applicable to the majority of cases met in an urban and non-urban polluted area can be used to evaluate the dust deposition-related derating factor (efficiency loss), which is very much sought after by the system designers, and tools used for computer modelling and system malfunction detection.

## Introduction

Solar energy is one of the major sources of renewable energy; it is free and non-permeable and clean and has various applications—direct and indirect ones. With the current development, it is reasonable to convert solar energy directly into the most desirable type of energy—electrical energy using a photovoltaic cell. The efficiency of photovoltaic (PV) modules systematically increases and at the same time the manufacturing prices gradually decrease. Therefore, in recent years, a significant increase in the solar energy investment is observed (Primer 2009). The performance of the photovoltaic modules is affected by several environmental factors such as wind speed, ambient temperature, humidity, rainfall, incident solar radiation intensity and spectrum, dust deposition, pollution and shadowing in addition to many alterable and unalterable factors that can influence the PV system efficiency. Dust, soiling and pollutants are one of the environmental factors that depend on the geographical location and that may be categorised within a set of factors that cannot be changed and that may significantly influence the photovoltaic efficiency whether particles are scattered in the atmosphere or deposited on the module surface.

In any case, the above factors involve the scattering of solar radiation and reduction of solar radiation intensity that can reach the photovoltaic module surfaces. The PV module performance decrease influenced by dust constitutes a local phenomenon and may differ significantly from region to region (Hassan et al. 2016a; Hassan et al. 2017; see also literature in Fig. 3). In recent years, many research studies were performed, reporting the dust and pollution influence on efficiency degradation of the photovoltaic modules. The air pollution can be considered in urban areas due to the combustion of fossil fuels, vehicles, construction activities and the industry (Benatiallah et al. 2012; Pang et al. 2006).

A considerable number of researchers have devoted their work to studying the sources and composition of the dust grains from many different regions of the world (Styszko et al. 2017). It is already known that the dust composition may differ depending on the region (Fujiwara et al. 2011). In dry climates and deserts, the key source of the dust is the soil (Bi et al. 2013; Ta et al. 2004), while in big cities, it is dust deposited on surfaces derived from many different local sources (Sarver et al. 2013) (i.e. coal-fired furnaces, cars, factories). In general, in the desert and semi-arid areas, the amount of naturally deposited dust is very high. Additionally, a relatively strong correlation between the absorbing impurities and the seasons has been observed (Ta et al. 2004). Soiling effect which refers to the particulate contamination of the optical surfaces includes dust accumulation, as well as surface contamination by plant products, bird droppings, soot and organic species.

Sarver et al. (2013) reviewed articles from around the world and took into account the module type, geographical region and the duration of dust deposition. They concluded that many studies showed that considerable PV module performance variations depend on exposure time. McTainsh et al. (1997) found that the grain size of the dust, as well as the pollution coming from the vicinity, had a great influence on the deposition of dust on the module front surface. The authors found that depending on the size of the grains that are spread differently, small particles below 5 μm in diameter are widely spaced, while particles larger than 20 μm are dust deposits from local sources. The deposited particle size plays an important role in absorption, scattering and reflectance of the incident solar radiation. The larger particles also have a greater tendency to resuspension even at moderate wind speed and this promotes deposition of smaller-size dust particles. On the other hand, as was revealed (Weber et al. 2014), smaller particles can cause higher module performance degradation than large particle for the same particle mass. The effect of the particle chemical and physical properties on the photovoltaic module efficiency was studied by El-Shobokshy and Hussein (1993). In their work, different types (carbon, cement, limestone) and sizes of particles (diameter 5, 10, 50, 60 μm) were deposited on a module surface and the module power output was measured. They concluded also that smaller particles have a more deteriorating effect on module efficiency than large ones. The result shows that electrical power output in the case of cement particles and carbon particles with the dust deposition density of 25 g/m^2^ decreases by about 40 and 90%, respectively.

The wind causes removal of the deposited particles and this effect depends primary on the wind speed and module tilt angle. However, it also depends upon the dust particle diameter and the microstructure of the dust layer. A thin layer of a deposited particle on the module which has been installed horizontally cannot be removed easily even at a high-speed wind of > 50 m/s. Hinds (1999) demonstrated that wind cleaning is very ineffective for particles with a diameter smaller than 50 μm and the primary reason is the adhesion force of the particles which is considerably higher for small particles than the removal force. For this reason, the size of the particle can vary significantly with the time, which was shown by Biryukov (1996). The author examined a natural dust sample collected from the Negev, Israel, using particle microscope analyses. The largest particle size varied from 20 to 40 μm and covered more than 50% of the module surface, while the smaller and larger particles constituted a minority. The particle deposition thickness directly influenced the current-voltage characteristics of photovoltaic modules. Jiang et al. (2011) and Jiang and Lu (2015) conducted experiments with the use of artificial impurities of the size of 1–100 μm and found that particle deposition caused a significant decrease in the PV module short-circuit current, while it did not affect the module open-circuit voltage. They also concluded that with increasing dust deposition density from 0 to 22 g/m^2^, the performance decreased from 0 up to about 26% nearly linearly and that in order to keep system performance at a nominal level, it is important to remove dust from the surface regularly, and this is particularly important for the locations with high air pollution and dry areas.

The deposited particle characteristics exert an important influence on soiling-related photovoltaic module efficiency degradation. It was demonstrated in various research works that the dust soiling energy degradation impact depends on the physicochemical properties of the particle (Kaldellis and Fragos 2011; Kaldellis et al. 2011; Khatib et al. 2013; John et al. 2016; Sulaiman et al. 2011). In that research work, as much as 15 types of dust pollutants have been identified, of which 6 types, ash, limestone, sand, calcium carbonate, silica and red soil, have a more important effect on PV performance degradation than others. Unfortunately, most of these studies use artificial dust particles with physicochemical characteristics which are not adequate to the natural dust composition. Relevant dust properties include size, shape and weight, charge distribution, electrostatic, material composition, shape and chemical and biological properties.

As was demonstrated by Kaldellis and Kapsali (2011), the important issue in the analysis of the performance degradation as a result of the dust deposition is that depending on the region, dust composition can vary significantly, and these variations considerably affect the reduction of module efficiency. The authors analyse the natural dust layer with a deposited density of 0.1 and 1.0 g/m^2^ which causes the module efficiency degradation of about 0.15 and 0.4% (in absolute terms), respectively. At the same time, an ash layer deposition with a deposited density of 0.6 and 2.1 g/m^2^ causes only a 0.15 and 0.4% performance reduction, respectively. On the other hand, red soil and limestone with a deposition of density 0.1 g/m^2^ cause a decrease of as much as 0.5% in efficiency.

Because the dust deposition on the module surface reduces its efficiency, some authors (Mani and Pillai 2010; Tylim 2013) give a recommendation for a cleaning cycle (two or three times a year) of the module front surface. Tylim (2013) concluded that the efficiency of the regularly cleaned photovoltaic system increases considerably, i.e. by 9 to 26%, relatively and that it depends on the system size. In a study performed by Saidan et al. (2016), it was revealed that the average degradation rate observed for solar modules exposed to the dust was 6.24% for 1 day, 11.8% for 1 week and 18.74% for 1 month in Baghdad, the capital city of Iraq. Zaihidee et al. (2016) investigated the dust deposition density on the photovoltaic physical parameters. Their results demonstrate that 20 g/m^2^ of dust accumulated on the photovoltaic panel surface reduces the short-circuit current by 15–21%, open-circuit voltage by 2–6% and the module efficiency by 15–35%, whereas Hottel and Woertz (1942) reported already in 1942 (for solar heat collector) the energy average reduction in the USA at around 1% every month in a humid subtropical or humid continental climate for 30^o^ tilted angle collector.

An experimental analysis of the dust particles deposition on the photovoltaic modules surface focusing on the effect of the temperature gradient for different operating temperatures was performed by Jiang and Lu (2015). Their results demonstrated that the energy output increases with a range from 0.947 to 0.971 by the increase of the temperature gradient close to the module surface. Mekhilef et al. (2012) evaluate the influence of three different parameters—wind speed, humidity level and accumulated dust—on the performance of the photovoltaic cells. The results demonstrate that the dust has the most significant influence as compared to humidity and wind speed. It was also noticed that all these factors should be considered together and not separately during a study of cell efficiency. Otherwise, the effects of other considerable efforts are ignored. The tilt angle of the photovoltaic modules strongly influences dust deposition (Appels et al. 2012; Hee et al. 2012; Hegazy 2001) as well as the cleaning of the modules by rain and wind. It has been found that the accumulation of large particles decreases with the increasing tilt angle and that the relative concentration of fine particles increases. Appels et al. (2012) described an experiment conducted in Leuven, Belgium, on the photovoltaic tilted module with 60^o^. Their results show that the dust reduces module efficiency approximately 0.75% per month. Hee et al. (2012) provided information that the average daily loss in Singapore due to the dust accumulation is about 0.3% of energy produced by the module. The study was conducted during 33 days with variously tilted angles ranging from 0^o^ to 90^o^, despite the heavy rains during the conducted period. In addition to the tilt angle, the effect of azimuth angle was simultaneously considered by (Elminir et al. 2006).

In the course of research conducted by Mejia et al. (2014) during 108 rainless days, it was revealed that the soiling accumulation of dust degrades the daily energy 0.2% in California, and after a 1-year analysis, it was reported that the accumulated dust influence is higher during the summer season than other sessions, and it was observed that after rain during autumn, the module efficiency increased to 7.1%. A similar value was observed during spring in California, USA (Cabanillas and Munguía 2011). In a similar research study performed during 3 months in two cities in Mexico (Hermosillo and Sonoro), the power reduction due to accumulative dust ranged from 4 to 7% for silicon crystalline modules, while for amorphous silicon modules, it was significantly higher—it ranged from 8 to 13%. A comparison between monocrystalline and polycrystalline silicon power reduction for 1-year non-cleaning photovoltaic modules in Senegal was performed by Ndiaye et al. (2013). The results show that the power reduction for monocrystalline modules is about 17.75% and that the reduction of polycrystalline modules is about 18.02%. In 480 days, an experimental study performed by Roth and Pettit (1980) revealed that the heavy rain cleaning can restore the photovoltaic module efficiency almost to the maximum level. An experimental study performed by Klugmann-Radziemska (2015) for three crystalline photovoltaic modules with a 37^o^ tilted angle in Gdańsk, Poland, illustrates that the relationship between the thickness of the pollution layer and the module energy losses is linear and that the daily efficiency losses of the modules are about 0.8%.

Research conducted in Thailand and described by Ketjoy and Konyu (2014) showed that the influence of accumulated dust quantity on the photovoltaic module for different periods (30 and 60 days) is 260 and 425 mg/m^2^·d, respectively, and that the effect of the decrease in module electrical power was 3.50 and 7.28% for amorphous silicon, 2.96 and 5.79% for monocrystalline silicon and 2.83 and 6.03% for multi-crystalline silicon, respectively, according to the dust quantity. Zorrilla-Casanova et al. (2011) stated in their research work that the accumulated dust caused photovoltaic module energy loss of about 4.4% in Málaga, Spain, and for the long rainless periods, this value may rise up to 20%. They also note that even small rain can greatly improve the conditions of the operation of the module. Adinoyi and Said (2013) found that the output power reduction of the modules was approximately 50% due to accumulated dust after a 6-month no-cleaning period in the east of Saudi Arabia. In Egypt, Elminir et al. (2006) in their experimental study using the module with a 45^o^ tilted angle revealed the photovoltaic module efficiency degradation due to accumulated dust to be about 17.4% per month.

In the research, in the deposition of dust conducted by Cuddihy (1983), it was revealed that one of the important identified processes which play a key role in efficiency degradation was the cementing of the dirt, which takes place when high levels of pollutants occur together with high humidity. Air pollution is composed of organic and inorganic particles, which may contain insoluble or soluble salts. In periods of high atmospheric humidity, dust particles create thin films of insoluble salt solutions which behave like cement and generate a local “shadow” on the photovoltaic module surfaces. The ecological and economic aspects of using renewable sources of energy (especially PV) in Iraq were comprehensively discussed by Hassan et al. (2016a, b).

An experimental investigation of three types of PV modules (monocrystalline, polycrystalline and single-junction amorphous silicon) was done by Bashir et al. (2015) and Ali et al. (2016). The results show that the output power of modules varies linearly with the irradiance results depicting that the module efficiency decreases with an increase in module temperature and the amorphous silicon module has shown the highest performance ratio. In research carried out by Ali et al. (2015b), a micro-channel was used for cooling PV cells, which results in the PV cell surface temperature drop of around 15 °C and the power increase around 14%. In Bashir et al. (2014), the comparative performance evaluation for three different types of photovoltaic modules in Taxila, Pakistan, was studied. The experimental investigation was carried out for winter months and the electrical power output, efficiency and performance were calculated taking into account the module temperature as well as the solar radiation effect. It was found that the average module efficiency decreased by about 8.8, 4.5 and 26% for monocrystalline, polycrystalline and single-junction amorphous silicon modules, respectively, with the module temperature increasing from 22 up to 33 °C.

Generally speaking, one may infer from this survey of literature the following conclusions: the environmental conditions and the geographical site with seasonal variations have a direct influence on the amount of dust and physical properties such as morphology, composition, gradation and mechanical properties. The roughness percentage and physical and chemical properties of the photovoltaic module surface (front side) have a significant influence on the dust deposit, causing a decrease in efficiency. The effect of air pollution is significant in urban areas due to the high population density, vehicles and growth in the industrial activities, specifically dust particles which are produced by the combustion of fossil fuels. Dust deposition on the front module surface can significantly reduce the amount of solar energy eventually absorbed by the module and changes in module output voltage and current are expected. The dust deposition has a very local effect and is strongly correlated with the air pollution. Due to this fact, it is very difficult to create a general model for dust deposition and dust-related PV system efficiency reduction. The properties of deposited dust and its impact on photovoltaic module performance are a complex task and they depend primarily on very localised environmental conditions. Much of the information available in the literature applies only to the specific location in which the experimental work was conducted. The important observation from literature led us to a critical point that large areas of the world still were not covered by the research. Additionally, a large number of research use artificial dust particles which are not adequate to the natural dust composition or use significantly different from natural artificial laboratory conditions. It is only through a systematic study of the dust accumulation at different locations of the Earth that the dust deposition effect on the PV power degradation can be better understood.

In this study carried out in Kraków, Poland, which is one of the most polluted European cities, the experimental investigation concerning the impact of natural dust deposition effect on the PV module front cover surface in the urban air-polluted area on the of photovoltaic system performance was studied. Most of the field studies report energy yield loss vs. exposure time without the detailed information about dust concentration density and this does not enable direct linking of a local pollution condition with the module performance degradation. In the present research, the mass of deposited dust and the photovoltaic module output power were experimentally measured in a natural high-pollution city environment during the heating and non-heating seasons for the various environmental conditions. It was intended to characterise dust accumulation on the photovoltaic module surface through a systematic approach.

The main objective of the present study is to develop a practical model for the simulation of the degradation of system efficiency (derating factor) caused by natural dust deposited on the front surface of the PV module. On the basis of an experimental measurement of natural dust deposition carried out by the authors in this study as well as on the basis of the scanty literature data for natural dust, an attempt was made to develop a reliable model which takes into account the major effects that dust deposition has on the performance of a PV system.

The proposed theoretical dust mass-dependent model for low and moderate naturally deposited dust concentration (up to 1 and 5 g/m^2^ and representative for many geographical regions) is applicable to the majority of cases met in an urban and non-urban polluted area and it can be used to evaluate the dust deposition-related derating factor which is very much sought after by the system designers, and tools used for computer modelling and system malfunction detection.

## Experimental setup and methodology

**Fig. 1:**
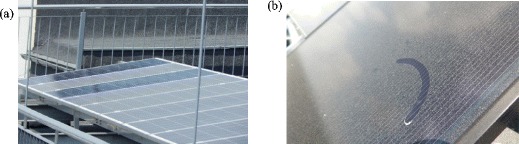
The PV modules directly used for analysis (**a**), and an example of dust deposition (**b**)

The influence of dust and soiling on the natural outdoor exposure of a PV system was conducted experimentally on the roof of the Faculty of Electrical Engineering, Automatics, Computer Science and Biomedical Engineering, AGH University of Science and Technology, in the city centre of Kraków, Poland (50.066354 N, 19.918191 E) characterised by high air pollution, high traffic intensity and relatively low wind speed. A PV system consists of two independent module strings connected in series. Strings are built by means of Sharp ND-RJ260-type polycrystalline photovoltaic modules with a nominal power of 260 W and *A*_*c*_ = 1.6 m^2^ of surface area (excluding the aluminium frame). During the experiment, the panels were adjusted at tilt angle *β* = 15^o^ and azimuth *γ* = 20^o^ West. In Fig. 1a, a part of the analysed photovoltaic system which consists of a total 21 photovoltaic modules in two independent strings is presented. Ten PV modules in the first string are equipped with individual optimisers (SolarEdge, P405, MPPT range 12.5-105 V) which monitor electrical parameters (current, voltage and output power) of the module and search for maximum power point *P*_max_ on every single module. The second string consists of 11 PV modules of the same type but all modules in this string are optimised at once using single-MPPT devices integrated with a PV inverter. The electrical parameters from the individual tracked PV modules and from the second string were acquired every minute and the average value was recorded for analysis every 15 min. The experimental procedure was carried out for all environmental conditions (clear sky, partially cloudy, overcast, rainfall) in the period 1.05–11.11.2017. For the current analysis, only the data from selected modules of the first string P_1–P_10 were presented and the data for the second string (denoted as module P_S with the average power normalised by the number of modules in a string *n* = 11). On the basis of the recorded measurements, the power output *P*_max_ of the polluted and clean PV modules was determined. At the same time, the dust mass deposited on the polluted module front surface was specified for each examined case. It is assumed that all modules experienced the same instantaneous (external) insolation level, ambient temperature or wind speed, as well as every single module is polluted uniformly, which means that all polycrystalline cells in the module are covered by the same dust mass.

The modules P_1 and P_3 were cleaned regularly and considered as reference modules, while the other modules were made dusty because they collected dust particles from polluted air in a natural manner. For the efficiency analysis, the dusty and clean modules were used for the purposes of reference in order to calculate efficiency loss. Afterwards, the polluted modules were also cleaned. In Fig. 1b, the example of the dust deposited on PV modules located nearby is presented. The dust deposited on the PV module surface (front cover—rough glass) was removed from the surface by means of distillate water and specially designed devices equipped with a scratcher and a suction pump. In order to remove the dust from modules, every single module was removed three times and the estimated (by cleaning the module six times) cleaning efficiency was about 86%. The dust water solution was collected in plastic containers, followed by freeze-drying (Liophilizator Alpha 1-4 LD). The mass of dust deposited on the surface of the modules after a specified time of exposure was evaluated gravimetrically. The OHAUS Discovery DV215CD balance with an accuracy of ± 0.01 mg was used for weighing. The difference in the values of mass plastic containers before *M*_*b*_ and after *M*_*a*_ sampling is the total mass of dust deposition on the surface of the polluted module *M*_*d*_. Finally, the dust deposition density *m*_*d*_ was estimated as follows:

1 $$ {M}_d={M}_a-{M}_b\kern1.25em {m}_d=\frac{M_d}{A_c} $$

The meteorological data (rainfall) were obtained by means of the Faculty of Physics and Applied Computer Science—the Vaisala WXT520 automatic meteorological station placed nearby was used. The concentration of PM10/PM2.5 was obtained from the Voivodeship Inspectorate of Environmental Protection in Kraków at an urban background station (al. Krasińskiego Station). Total suspended particles (TSP) were collected at the PV system site. A low-volume sampler was placed on the roof next to the PV modules. Samples were collected on quartz fibre filters (Pallflex, Pall Life Sciences) of a 47-mm diameter. On weekdays, the filters were replaced at 24 ± 2 h intervals and during weekends after around 70 h. After the data acquisition and the calculation procedure, the total errors of the measurements were determined by analysing the accuracy of the equipment.

## Results and discussion

### The dust deposition analysis

**Fig. 2:**
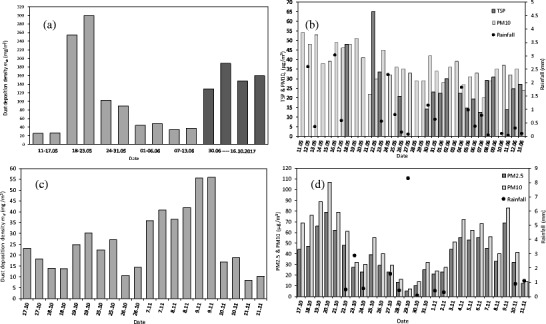
The dust deposition density at the PV module surface (**a**, **c**) and TSP, PM10, rainfall or PM2.5 (**b**, **d**)

The dust samples were collected from cleaned modules on the basis of a daily to weekly schedule and were the subject of analysis. They represent various exposure times of the dust deposited at the module surface and were collected regardless of the weather conditions during past days (rains, low/high pollution, strong wind). The weekly dust deposition density *m*_*d*_ for five consecutive weeks (in the period 1.05–30.06.2017) for two identical modules (P_1 and P_3) is presented in Fig. 2a. One may infer that the weekly mass deposition differs by up to 15% between identical successively cleaned PV modules as well as their mass varies week by week more than 11 times and the lowest weekly dust deposition density *m*_*d*_ was 25.8 mg/m^2^ while the highest was 300.0 mg/m^2^. Due to the stochastic nature of variations of the environmental condition including pollution, the dust deposition imparts this nature. The total weekly dust mass deposited at the PV module surface depends on the air pollution, wind speed, humidity and—what is most important—rainfalls which occurred during the period of analysis almost every week. The only week without rainfall was recorded for the week 17–23.05.2017 when the highest dust deposition density was observed. In this figure, 15 weeks, dust deposition density (in the period 30.06.2017–16.10.2017) is presented also. One may see from Fig. 2a that a much longer exposure time does not cause higher dust deposition density *m*_*d*_ and what is also worth noting is that a longer time causes a significant difference from 9 up to 41% in the mass deposition density between identical modules (P_2, P_5, P_8, P_10). In Fig. 2b, the TSP concentration, particles matter concentration with aerodynamic diameter below 10 μm (PM10) and rainfall are presented for reference. During the analysis, one observed a large variability in the dust deposition, which manifested its dependence primarily on the environmental conditions and secondarily on the exposure period.

In order to analyse dust deposition under more stable conditions, an exposure time as short as 1 day for dust collection was applied. In that case, dust samples were collected from modules (P_1 and P_6 or P_8) every day during the period 17.10–11.11.2017. The daily dust deposition density *m*_*d*_ for two identical modules is presented in Fig. 2c. One may see that the daily deposition between the pairs of identical modules differs in the range 0.5–25%. One should notice that most of the results for the 1-day exposure period were obtained for the days without rainfall, which may be inferred from Fig. 2d. Generally speaking, dust deposition density *m*_*d*_ for rainless days follows particle concentration in the air but even for rainless days for the comparable value PM10 (17–19.10 and 7–9.11), the deposition differs by up to 125%. The reasons for this are associated with other environmental parameters, for example, water condensation at module surface (on 18.10 and 8.11) or very low wind speed (on 9.11). More information about daily to weekly dust deposition rate as well as correlation with environmental variables or chemical dust composition analysis for the presented location can be found in other works of the authors (Styszko et al. 2018).

**Fig. 3:**
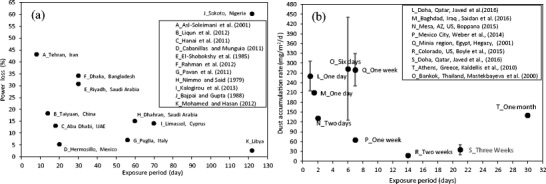
The PV module power loss (**a**) and dust accumulation rate (**b**) vs. exposure period presented in reference to different sites by different authors

As we have observed, the dust deposition density is time-dependent (Fig. 2) but even for the rainless period for as short a duration as 1 day (Fig. 2c), this parameter differs significantly. The exposure time does not provide sufficient information about solar radiation attenuation due to dust deposition. The system efficiency loss vs. time which is reported in literature (Jiang et al. 2011; Kaldellis et al. 2010; Ali et al. 2015a) can be as large as 77% or as small as 9%, and what may be inferred from Fig. 3a (Asl-Soleimani et al. 2001; Liqun et al. 2012; Al Hanai et al. 2011; Cabanillas and Munguía 2011; El-Shobokshy et al. 1985; Rehman and El-Amin 2012; Pavan et al. 2011; Nimmo and Said 1981; Kalogirou et al. 2013; Bajpai and Gupta 1988; Mohamed and Hasan 2012). The module or system maximum power *P*_max_ losses vs. time are not really correlated with the time. It became clear that the dust exposure time in the natural environment is not a relevant parameter. This type of studies may provide quite important knowledge related to the local dust deposition rate. However, in the majority of research studies of this type (Jiang et al. 2011; Kaldellis and Kapsali 2011; Ali et al. 2015a), dust mass deposition is not available.

Additionally, most of the detailed studies available in the literature were performed by means of artificial dust particles (Sayyah et al. 2014; El-Shobokshy and Hussein 1993; Kaldellis and Fragos 2011; Kaldellis et al. 2011) and a few studies used natural dust (Kaldellis et al. 2010; Ali et al. 2015a) deposited on the surface of the PV module. On the other hand, in many other studies, the focus is on the physical and chemical analysis of dust particles (Kaldellis et al. 2011; Tian et al. 2007; El-Shobokshy and Hussein 1993), the effect of particle deposition on solar beam attenuation (Kumar et al. 2013; Ketjoy and Konyu 2014) or on the dust accumulation rate (Javed et al. 2017; Saidan et al. 2016; Boppana 2015; Mastekbayeva and Kumar 2000; Weber et al. 2014; Hegazy 2001; Boyle et al. 2015; Kaldellis et al. 2010), but unfortunately, most of the data (Fig. 3b) fail to take power losses into account.

Due to this fact, at the current stage, it is not possible to use the huge number of data which is available in the world (Fig. 3) to create a universal model of practical utility.

At this point, it seems to be clear that the PV efficiency loss due to dust deposition and soiling should be correlated with the dust mass deposition or, generally speaking, with the dust deposition density (on the PV module front surface) and not with the time—as was reported in a large number of research studies (Fig. 3a). Even for a very long time scale, this information is hardly useful for PV system analysis due to decorrelation of the data, particularly the unpredictable decorrelation of the mass deposition with the solar radiation intensity.

According to the authors, in order to evaluate photovoltaic module efficiency degradation due to dust effect and to perform a reliable PV system analysis, two quite independent models are required. The first model must be able to correlate the amount of dust deposited on the module surface with photovoltaic module efficiency loss and the second one which correlates the amount of dust with locally available weather or environmental conditions (the geographical location of the site). Model splitting will allow distinguishing various climate conditions, from different geographical parts of the world with diverse climatological conditions, including those of semi-arid zones or deserts and different climate zones. In order to create a theoretical model of dust mass deposition, an experimental measurement of air pollution and local weather conditions as well as dust particle properties is highly desirable for different world locations, which is far beyond the scope of this paper.

In the next section, the effect of the dust deposition on the photovoltaic module efficiency under natural conditions is presented together with the dust deposition-related theoretical model for predicting photovoltaic system efficiency loss (derating factor) caused by dust deposited on the module surface.

### The effect of dust deposition on the efficiency of the PV module

In order to properly design and optimise the photovoltaic or photovoltaic base hybrid system, the PV module-generating electrical power *P*_*PV*_ has to be calculated on the basis of the module specification and solar radiation prediction or historical local real measurement. The PV module power depends on the total solar irradiance incident on the PV surface (normal component) *G*_*T*_ but it also depends on several additional factors such as dust and soiling, losses, shading, age, snow cover and temperature. In the literature, a large number of formulas for PV power output *P*_*PV*_ of varying complexity were proposed (Duffie and Beckman 2013; Hay 1986; Reindl et al. 1990). One of the most practical applications can be written as follows (Hassan et al. 2017):

2 $$ {P}_{\mathrm{PV}}={Y}_{\mathrm{PV}}{\eta}_{\mathrm{der}}\left[1+{\alpha}_p\left({T}_c-{T}_{c,\mathrm{STC}}\right)\right]\left(\frac{{\overline{G}}_T}{{\overline{G}}_{T,\mathrm{STC}}}\right)={\overline{G}}_T\cdot {A}_C\cdot {\eta}_{\mathrm{PV}}\cdot {\eta}_{\mathrm{der}}\left[1+{\alpha}_p\left({T}_c-{T}_{c,\mathrm{STC}}\right)\right] $$

where *Y*_PV_ (W) is the rated capacity of the PV module (power output under STC conditions), *η*_der_ (%) is the PV module derating factor, *G*_*T*_ (W/m^2^) is the total incident solar irradiance*, α*_*P*_ (%/°C) is the PV temperature coefficient for the power, *T*_*C*_ (°C) is the PV module instantaneous temperature. *T*_*C*,STC_ (°C) and *G*_*T*, STC_ (W/m^2^) are the PV module temperature and solar irradiance at standard test conditions and *A*_*C*_ (m^2^) is the module area and *η*_PV_ (%) is the module efficiency.

The PV module derating factor is introduced taking into account additional factors affecting the module such as dust and soiling, shading, age and snow cover and can be decomposed into few key derating components and written as follows:

3 $$ {\eta}_{\mathrm{der}}={\eta}_{\mathrm{dust}}\cdot {\eta}_{\mathrm{shade}}\cdot {\eta}_{\mathrm{age}} $$

where *η* (%) are the module derating factors caused by the dust and soil *η*_dust_, shading *η*_shade_ and age *η*_age_.

To the best of our knowledge, in the literature, no work has been done to correlate the derating factors *η*_dust_ to the amount of natural dust deposition on PV module surface. When we explored a large number of papers, it turned out that only four papers (Gholami et al. 2018; Ali et al. 2015a; Sayyah et al. 2014; Kaldellis and Kapsali 2011) provide the data in question (three for natural pollution and one for artificial pollution) which can be directly used as a basis for the development of a theoretical model. All the relevant data available in the literature will be subsequently used to validate and extend the range of the proposed model’s functionality and will be discussed at the end of this chapter.

**Fig. 4:**
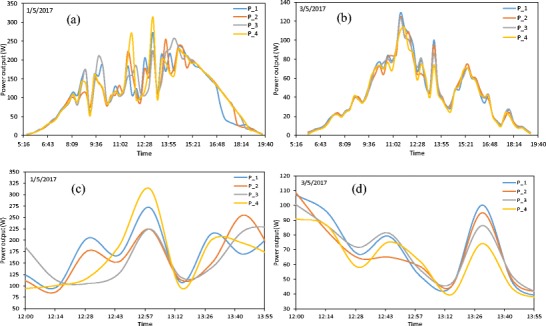
The average power output for four modules P_1, P_2, P_3, and P_4 at sunny (**a**) and cloudy (**b**) days and the zoom for the middle of the day (**c**, **d**)

In order to evaluate the dust mass deposition-dependent theoretical model for derating factors *η*_dust_, the maximum power output *P*_max_ at module characteristic maximum power point from all analysed PV modules and the mass deposition were acquired. The mean *P*_max_ was recorded every 15 min on the basis of a 1-min acquisition system sampling rate. Because the optimisers required power to work and this power is taken from the modules, the recording time was always from sunrise until sunset. The average maximum power output *P*_max_ for the four modules P_1–P_4 for the period which preceded the procedure of cleaning is presented in Fig. 4 for the sunny day 1.05.2017 and the cloudy day 3.05.2017. The results show that even though the modules are of the same type, they deliver an unequal amount of the power. Assuming the modules are subject to exposure to the almost identical weather conditions (solar radiation, air temperature) and geometrical conditions (the same angle, reflections), the difference in the obtained power output from modules could be attributed to the manufacturing process. It is obvious that even the modules from one batch line and series are not completely identical. The power difference due to the manufacturing process is small and the maximum tolerance in peak power *P*_PVmax_ for this type of modules is up to 3% (the datasheet of Sharp ND-RJ260 module can be found at www.sharp.eu). However, as far as the same module series and batch line are concerned, this tolerance is usually not higher than 1%.

As can be seen in Fig. 4, the power output *P*_max_ from modules is continuously fluctuating, but what is more important is that the difference in acquired power output *P*_max_ between the analysed modules is not constant. This effect can be better seen in Fig. 4c, d where the time scale was magnified for the sake of clarity. As was already mentioned, the dust layer growth at the module surface may differ slightly from module to module. The weekly maximum difference observed in dust layer (Fig. 2a) is about 15% and the weekly average efficiency loss for a country with similar dust conditions—Spain—is about 1%, as mentioned by Klugmann-Radziemska (2015). The calculated maximum power variation due to this effect should be below 0.45 W and not about 65 W as observed for some time periods in Fig. 4. The “unpredictable” fluctuations in the power output can be only explained due to the optimiser algorithm device (SolarEdge, P405) which continuously searches for the maximum power point (MPPT algorithm) for each individual module. The tiny difference between the modules can cause significant instantaneous power variation and this variation is due to the use of independent (asynchronous) optimisers among modules. For this reason, the instantaneous power output cannot be directly used for the analysis and the relatively long period of averaging (1 day) has to be used to smooth fluctuation and to evaluate the dust deposition effect.

**Fig. 5:**
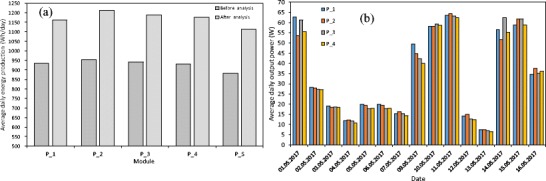
The average daily energy production (**a**) and average daily power output (**b**) for modules P_1–P_4 and one string P_S

In Fig. 5a, the average daily energy production for modules P_1–P_4 is presented. The energy was calculated for different environmental conditions (solar radiation intensity) on the basis of an analysis of 10 days which precedes the cleaning of modules (denoted as before the analysis) and on the basis of the analysis of 5 days when the cleaning procedure was finished (denoted as after the analysis). It can be observed that the energy production for various PV modules differed significantly mainly due to a slight difference in nominal power and a tiny difference in the environmental conditions. In order to account for the effect of nominal power output difference, a power output *P*_max_ normalisation procedure is implemented and the module correction factor is evaluated on the basis of the energy yield *E* of the PV module for the period of time (1 day):

4 $$ {\kappa}_i=\frac{E_i}{E_1}\mathrm{and}\kern0.5em {E}_i={\int}_{\left({t}_o\right)}^{\left({t}_o+\Delta t\right)}{P}_{\mathrm{max}}(t) dt $$

The calculated mean correction coefficient *κ* for modules P_1–P_4 is presented in Table 1.

**Table 1:** Module power correction coefficients

Correction coefficient *κ*	Module P_1	Module P_2	Module P_3	Module P_4
Mean	1.0	0.958	0.978	0.989

In Fig. 5a, the average daily energy production for a second string which consists of module P_S is also presented. In this string, no individual optimisers are presented and all 11 modules are optimised at once using MPPT algorithm built in into a single inverter. One may infer from Fig. 5a that the average daily electrical energy production $\overline{E}$ for module P_S is about 5.5–11.2% lower than that for the modules with individual optimisers. This may justify installation of individual optimisers for each module. The average daily power output ${\overline{P}}_{\mathrm{max}}$ for modules P_1–P_4 and for the selected days is presented in Fig. 5b. The average power output variation is significant between the modules on the same day as well as on the different days due to significant variations in incident solar radiation.

**Fig. 6:**
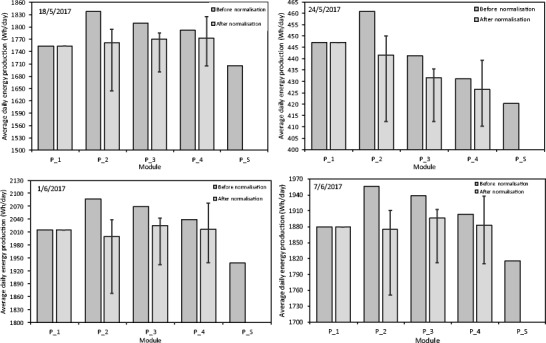
The daily average energy production (before and after normalisation) with uncertainty for modules P_1–P_4 and P_S

In Fig. 6, the average daily energy production $\overline{E}$ together with normalised average daily energy production ${\overline{E}}_n=\kappa \bullet \overline{E}$and with uncertainty for the day next after the cleaning and for modules P_1 and P_3 is presented (the remaining PV modules P_2 and P_4 are left with the dust layer for reference). It can be seen that for the first day of the analysis (18.05.2017) when the dust deposition density was very low—*m*_*d*_ = 25.8 mg/m^2^ (see Fig. 2), the normalised daily energy production for the clean modules was even slightly lower than that for the modules with the dust layer. This suggests the small positive effect of the tiny dust layer. Typically, PV modules are not covered by the flat glass surface but a specialised glass with significant surface roughness and a special texture is used in order to achieve better light trapping and absorption in solar cells. Moreover, it is possible that the tiny dust surface which virtually increases surface roughness may have a positive effect. However, one should notice that the effect is at the margin of the measurement uncertainty (caused mainly by the power output fluctuation and the dust collection methodology). When the natural dust deposition mass on polycrystalline photovoltaic modules grew, then the normalised average daily energy production decreased. The highest recorded deposited dust density was *m*_*d*_ = 300.0 mg/m^2^ (24.05.2017) and this amount is responsible for the decrease in energy production of about 2.1%. For the other days (and masses), the observed effect is also clearly perceptible. For all the days, a significantly lower electrical energy production was observed for module P_S and the effect is primary caused by the system optimisation and secondarily by the dust deposited on the surface (in the case of P_S modules, dust is removed only with the natural environmental processes).

During the testing period, in the majority of cases, the power output delivery by the modules decreases due to dust accumulation. Since, generally speaking, the power rating can be different for the different module types and sizes, in order to calculate module performance loss, normalised results should be taken into consideration. Typically, in the literature (Ali et al. 2015a; Zaihidee et al. 2016; Sayyah et al. 2014), the module efficiency, reduction in power output and reduction in module efficiency are presented and the last parameter is sometimes normalised and sometimes not and what is more important is that the percentage efficiency reduction is presented in absolute terms (percentage minus percentage) or otherwise, which is very confusing:

5 $$ {\Delta}_{P_{\mathrm{loss}}}=\frac{P_c-{P}_d}{P_c}\bullet 100\% $$

6a $$ {\Delta}_{\eta_{\mathrm{loss}}}=\frac{\eta_c-{\eta}_d}{\eta_c}\bullet 100\% $$

6b $$ {\Delta}_{\eta_{\mathrm{loss}}}^{\ast }={\eta}_c-{\eta}_d $$

where *P*_*c*_ and *P*_*d*_ are the (maximum) power output of the clean and dusty modules while *η*_*c*_  and *η*_*d*_ represent the efficiency of the clean and dusty modules calculated from the following equation:

7 $$ {\eta}_{\mathrm{PV}}=\frac{P_{\mathrm{max}}}{G_T\bullet {A}_c}\bullet 100\% $$

When we implement Eq. (7) to Eq. (6a) and use Eqs. (5) and (4), it becomes clear that all properly normalised losses for the averaged value will become equal and can be evaluated from the average maximum power output ${\overline{P}}_{\mathrm{max}}$ or energy $\overline{E}$:

8 $$ {\Delta \eta}_{\mathrm{loss}}=\frac{{\overline{P}}_c-{\overline{P}}_d}{{\overline{P}}_c}=1-\frac{{\overline{P}}_d}{{\overline{P}}_c}\ \mathrm{or}\ {\Delta \eta}_{\mathrm{loss}}=1-\frac{{\overline{E}}_d}{{\overline{E}}_c} $$

where ${\overline{P}}_d\kern0.75em \mathrm{and}\ {\overline{P}}_c$ (W) and ${\overline{E}}_d\kern0.75em \mathrm{and}\ {\overline{E}}_c$ (Wh) are the mean power and mean energy output from dirty and clean modules respectively, and the last term in Eq. (8) can be defined as a dust-related derating factor used in Eq. (3):

9 $$ {\eta}_{\mathrm{dust}}=\frac{\eta_d}{\eta_c}=\frac{{\overline{P}}_d}{{\overline{P}}_c}=\frac{{\overline{E}}_d}{{\overline{E}}_c} $$

**Fig. 7:**
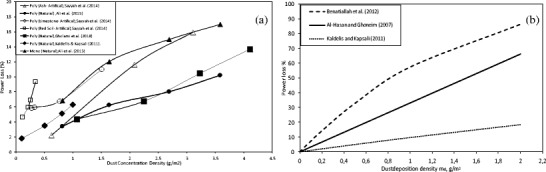
The average power loss due to the natural (open symbol) or artificial (fill symbol) dust deposition based on literature data (**a**) and theoretical models (**b**)

Most of the investigations presented in the literature were conducted in arid and semi-arid areas and they were performed using artificial dust. Only a few studies deal with natural dust. The key and unique data available in the literature (Gholami et al. 2018; Ali et al. 2015a; Sayyah et al. 2014; Kaldellis and Kapsali 2011) are presented in Fig. 7a. Additionally, in the literature data, only the results of Ali et al. (2015a) are presented for two different types of modules: mono- and polycrystalline, while almost all other studies concentrate on one type of PV technologies only. All relevant data from the literature for natural dust deposition will be used to develop the theoretical model which is proposed later on in this paper. In the literature, three theoretical models (Benatiallah et al. 2012; Al-Hasan and Ghoneim 2007; Kaldellis and Kapsali 2011) which correlate dust concentration density with the PV module power losses can be found. Most of them are based on experiments conducted by means of artificial dust (red soil, ash, sand, silica, calcium carbonate, limestone, carbonaceous fly ash particles).

The key point which may be drawn from a review of models is that there is no theoretical model applicable to all the pollutant types and different PV types. Additionally, the models represent specific and particular cases of a narrow range of functionality.

According to the experimental measurement presented in Fig. 7a performed for different dust types, the maximum power loss caused by the dust deposition equal to 1.0 g/m^2^ should not exceed 8%, while the power loss predicted by the Benatiallah et al. (2012) model or the Al-Hasan and Ghoneim (2007) model (Fig. 7b) overestimates this value four and eight times, respectively. What is more important is that this discrepancy increases with the dust deposition density. This leads us to conclude that there is still a necessity of a relevant model which can be used to calculate the effects of dust.

On the basis of the information presented above, the current study is focused on the investigation of the natural dust impact on the polycrystalline PV module efficiency loss (derating factor) in the urban environment and, with the help of literature data, also for other environments and module types. The key aim is to evaluate the relation between the derating factor *η*_dust_ (Eq. (9)) and the dust deposition density *m*_d_ by means of experimental measurements and the data available in the literature (Fig. 7a).

**Fig. 8:**
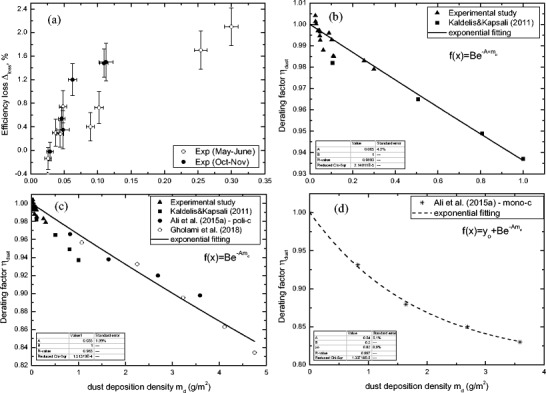
The average energy loss due to the natural dust deposition: current experimental measurement (**a**), theoretical model up to 1 g/m^2^ (**b**), theoretical model up to 5 g/m^2^ (**c**) and for monocrystalline modules (**d**)

According to the experimental results which were obtained, the measured dust deposition density *m*_*d*_ ranged from 0.0258 to 0.300 g/m^2^. The effect of dust deposition *m*_*d*_ in the periods May–June and October–November on the efficiency loss is presented in Fig. 8a. Moreover, there is a significant error in efficiency evaluation—one may infer from this figure that efficiency loss is not only mass-dependent. For similar or even larger masses, the effect on efficiency varies from day to day. This suggests that the deposited dust structure (type and size) is also important. The dusts from the same period have similar properties and the effect of mass is a key issue while the dusts from different periods or seasons have different properties and the dust mass is not the only key factor. On the other hand, the analysis performed on the same day but for two different modules confirms that power increases when the dust deposition is very small 0.0258 and 0.026 g/m^2^. This phenomenon is not observed for higher deposition.

In order to develop a practical relation, an exponential function has been selected as the most appropriate among other functions. Due to relatively moderate pollution and rainfalls, the range of the dust deposition density is relatively narrow (nevertheless, data can provide useful information). In order to extend the model range, all current experimental measurement points as well as experimental measurement for natural dust available in the literature (Kaldellis and Kapsali 2011) are used and presented in Fig. 8b. One may infer from this figure a significant decrease in the derating factor (increase in efficiency loss) observed when the dust deposition density increases. The efficiency reduction gradually increased with the mass and it follows the nonlinear trend. Having taken into consideration all measurement point and error bars, as well as experimental measurement for natural dust (four points; Kaldellis and Kapsali 2011) available in the literature, the exponential fitting curve has been created representing the model for derating factor vs. dust deposition density *m*_*d*_. The theoretical model (Eq. (10)) with the coefficients presented in Table 2 was calculated for the analysed system configuration (polycrystalline photovoltaic modules). The correlation of the fitting line with the results is deemed to be sufficiently reliable with the *R* square close to 0.98. The model is moderately dependent on environmental conditions, and after adaptation, it can be used in any location to calculate the PV derating factor vs. dust density deposited at the module surface:

10 $$ {\eta}_{\mathrm{dust}}={Be}^{-A\bullet {m}_d} $$

**Table 2:** Evaluated model constants

Symbol	*A*	*A* error (%)	*B*
Model: poly PV up to 1.0 g/m^2^	0.068	4.20	1.0
Model: poly PV up to 5.0 g/m^2^	0.035	1.89	1.0
Model: mono PV up to 5.0 g/m^2^	0.54	5.1	0.20

where *A* and *B* are the model constant and *m*_*d*_ is the mass deposition density, g/m^2^.

This trend line can be even extended up to 5.0 g/m^2^ (Fig. 8c) by means of natural dust experimental measurement (in Taxila, Pakistan) provided by Ali et al. (2015a) and (in Tehran, Iran) by Gholami et al. (2018). For this wide range (from 0 up to 5 g/m^2^), coefficients are different.

The ranges up to 1.0 or 5.0 g/m^2^ represent low and moderate dust depositions. The higher dust concentration is usually artificially dispersed on the PV module surface and higher values seldom occur in real cases. For most cases, the module will be cleaned by the rain, and excluding very unique cases (sandstorms), it is hardly possible to obtain natural dust concentration higher than 5.0 g/m^2^.

In order to calculate dusty module efficiency *η*_*PV*_ loss having nominal *η*_*o*_ STC module efficiency (clean one), the following practical relationship can be used:

11 $$ {\Delta}_{\eta_{\mathrm{loss}}}={\eta}_o\left(1-{Be}^{-A\bullet {m}_d}\right) $$

Ali et al. (2015a) provided also experimental measurement for monocrystalline photovoltaic modules. By using those data, another theoretical correlation was created for monocrystalline photovoltaic modules with the coefficient presented in Table 2. As we may see for monocrystalline PV (Fig. 8d), now the derating factor decreases much faster, and for mass 1 g/m^2^, it is as low as 0.92, while for polycrystalline, it is about 0.95 and 0.97. This is more than what was observed for polycrystalline loss in efficiency which puts into question the benefits of the greater efficiency of monocrystalline photovoltaic modules.

It was already demonstrated that efficiency loss is not only mass-dependent but also PV type-dependent as well as dust type- and dust size-dependent. In order to take into account the dust type and type effect in Eq. (10), apparent dust deposition density ${m}_d^{\ast }$ instead of real deposition density *m*_*d*_ can be introduced:

12 $$ {m}_d^{\ast }={C}_s{C}_t\bullet {m}_d $$

where *C*_*s*_ and *C*_*t*_ are average size dust-dependent and average type dust-dependent correction coefficient. Natural dust type and size are local site-dependent and may follow seasonal fluctuation. It is important to notice that the average dust size deposited at the module surface varied with the time of deposition (Biryukov 1996). Due to particle-flow (wind effect) interaction, large particles are removed more effectively than small ones (Weber et al. 2014; Hinds 1999). This effect significantly influenced the average dust particle size and it can be accounted for by the variable correction coefficient *C*_*s*_. In order to evaluate correction coefficient, *C*_*t*_ information about local dust particle physical properties (Kaldellis et al. 2011; Khatib et al. 2013; John et al. 2016; Sulaiman et al. 2011) is required.

## Conclusions

The dust deposition effect on photovoltaic module performance is a complex problem and it depends primarily on very localised environmental conditions. It is only through the systematical study of the natural dust accumulation in different locations of the Earth and at different seasons that the dust deposition effect on the PV system degradation can be better understood. The dust accumulation on the photovoltaic modules involves energy production loss and thus a decrease in the overall photovoltaic system efficiency. It also generates economic loss. In this study conducted in the city centre of Kraków, Poland, characterised by high pollution and low wind speed, the focus is on the evaluation of the efficiency degradation (derating factor) of polycrystalline photovoltaic modules due to natural dust deposition. The experimental measurement was conducted under variable environmental conditions and under different dust deposition exposure periods. It was observed that the power output is significantly influenced by the dust deposition and the highest efficiency decrement observed in the current experiment was 2.1% (for maximum obtained dust deposition density equal to 300.0 mg/m^2^) which is a significant decrement, considering the fact that this mass was deposited during 1 week. The efficiency loss gradually increased with the dust mass deposited and it follows the exponential trend line. It was observed that this loss is not only mass-dependent but that it also depends on the dust properties. The samples from the same period have similar properties and the effect of mass is a key issue while the samples from different periods have different properties (dust deposition under different environmental conditions—wind, humidity, rainfall), and in that case, the sample mass is not the only but still a key factor. On the basis of the results which were obtained, one may state that the particles which exist in the air of polluted urban areas negatively affect the PV module performance and the level of degradation is non-negligible. The derating factor (efficiency loss) depends on the apparent mass of the dust particles deposited on the module surface and PV module type. The performance of PV module can be considerably reduced even after a small exposure time into the atmospheric air (more than two percentage point in power loss in 1 week).

The experimental results enable the creation of a simple but reliable mathematical model (Eqs. (10)–(12)) for the performance degradation caused by dust mass deposited on the front cover surface of PV modules. Having taken into consideration all measurement points as well as experimental measurement for natural dust available in the literature (Kaldellis and Kapsali 2011), the exponential fitting curve has been created representing the model for derating factor vs. dust deposition density *m*_*d*_*.* The theoretical model (Eqs. (10)–(12)) with the coefficient presented in Table 2 was calculated for the analysed system configuration (polycrystalline photovoltaic modules). This trend line has been extended up to 5.0 g/m^2^ by means of natural dust experimental measurement provided by Ali et al. (2015a) (in Taxila, Pakistan) or Gholami et al. (2018) (in Teheran, Iran). For this wide range—from 0 up to 5.0 g/m^2^—coefficients are different. The ranges up to 1.0 or 5.0 g/m^2^ represent low and modest dust depositions. The higher dust concentration is usually artificially dispersed on the PV module surface and values which are higher than that seldom occur in real cases. The maximum performance loss caused by the dust deposition density equal to 1 g/m^2^ predicted by the proposed theoretical models is in the range of 6–8% which is consistent with the data presented in the majority of research works. The small positive effect of the tiny dust layer (slight increases in surface roughness) on the module performance was observed which results in better light trapping and absorption in solar cells but still without a significant decrease in light transmissivity.

With the use of the only experimental measurement for natural dust effect but for monocrystalline photovoltaic modules (Ali et al. 2015a), a different coefficient for theoretical correlation has been evaluated. As it was demonstrated for monocrystalline modules, the derating factor decreases much faster with the natural dust mass deposited than for the polycrystalline one. This significant loss in efficiency puts into question the benefits of greater efficiency of monocrystalline modules.

It was proposed that in order to evaluate photovoltaic module efficiency degradation due to the dust effect and to perform a reliable PV system analysis, two independent models are required, the first model capable of correlating the amount of dust deposited on the module surface with photovoltaic module efficiency loss and the second one correlating the amount of dust with locally environmental conditions. Model splitting will enable the distinguishing of various climate conditions, from different geographical parts of the world with diverse climatological conditions including semi-arid areas or deserts and different climate zones. In order to create dust mass deposition theoretical model, a more detailed analysis from different locations in the world is required, which is far beyond the scope of this paper. This type of study could be the main subject of investigation for groups of researchers who represent other fields of study.

The proposed mass-dependent model for low and moderate naturally deposited dust concentration can be used to evaluate dust deposition-related derating factor (efficiency loss) which is much sought after by the system designers and tools used for and computer modelling of hybrid energy systems or to detect system malfunction (low performance) due to dust deposition. This relation can help to predict the effect of dust deposition on PV module performance in real environmental conditions.
